# Risk factors and short-term outcomes of postoperative pulmonary complications after VATS lobectomy

**DOI:** 10.1186/s13019-018-0717-6

**Published:** 2018-04-12

**Authors:** Paula J. Agostini, Sebastian T. Lugg, Kerry Adams, Tom Smith, Maninder S. Kalkat, Pala B. Rajesh, Richard S. Steyn, Babu Naidu, Alison Rushton, Ehab Bishay

**Affiliations:** 1 Department of Thoracic Surgery, Heart of England NHS Foundation Trust, Bordesley Green East, Birmingham, UK; 2 School of Sport, Exercise and Rehabilitation Sciences, University of Birmingham, Birmingham, UK; 3 Institute of Inflammation and Ageing, University of Birmingham, Birmingham, UK

**Keywords:** VATS, Lobectomy, Pneumonia, Atelectasis, Risk factors

## Abstract

**Background:**

Postoperative pulmonary complications (PPCs) are associated with poor outcomes following thoracotomy and lung resection. Video-assisted thoracoscopic surgery (VATS) for lobectomy is now frequently utilised as an alternative to thoracotomy, however patients remain at risk for development of PPC. There is little known of the short-term outcome associated with PPC following VATS lobectomy and if there are any potential risk factors that could be modified to prevent PPC development; our study aimed to investigate this.

**Methods:**

A prospective observational study of consecutive patients undergoing VATS lobectomy for lung cancer over a 4-year period in a regional centre was performed (2012–2016). Exclusion criteria included re-do VATS or surgery for pulmonary infection. All patients received physiotherapy as necessary from postoperative day 1 (POD1) and PPC was determined using the Melbourne Group Scale. Outcomes included hospital LOS, intensive therapy unit (ITU) admission and hospital mortality.

**Results:**

Of the 285 patients included in the study, 137 were male (48.1%), the median (IQR) age was 69 (13) years and the mean (±SD) FEV_1_% predicted was 87% (±19). Patients that developed a PPC (*n* = 21; 7.4%) had a significantly longer hospital LOS (4 vs. 3 days), higher frequency of ITU admission (23.8% vs. 0.5%) and higher hospital mortality (14.3% vs. 0%) (*p* < 0.001). PPC patients also required more physiotherapy contacts/time, emergency call-outs and specific pulmonary therapy (*p* < 0.05). Current smoking and COPD diagnosis were significantly associated with development of PPC on univariate analysis (*p* < 0.05), however only current smoking was a significant independent risk factor on multivariate analysis (*p* = 0.015).

**Conclusions:**

Patients undergoing VATS lobectomy remain at risk of developing a PPC, which is associated with an increase in physiotherapy requirements and a worse short-term morbidity and mortality. Current smoking is the only independent risk factor for PPC after VATS lobectomy, thus vigorous addressing of preoperative smoking cessation is urgently needed.

## Background

Lung cancer is the leading cause of cancer death in the UK [1]. Lobectomy is widely considered the optimal therapy for early stage non-small cell lung cancer (NSCLC) [2]. Video-assisted thoracoscopic surgery (VATS) is being increasingly performed for early-stage NSCLC instead of open thoracotomy because of its minimally invasive nature [3].

Postoperative pulmonary complications (PPCs) after major thoracic surgery, such as pneumonia and clinically significant atelectasis are common, and increase hospital mortality, intensive therapy unit (ITU) admission and hospital length of stay (LOS) [4]. Patients developing a PPC also have a worse long-term outcome; after thoracotomy and lung resection PPC resulted in a 6-month reduction in the mean overall survival (*p* = 0.006) [5]. Risk factors for developing PPC after thoracotomy and lung resection have been previously defined as age, smoking, chronic obstructive pulmonary disease (COPD), percentage predicted forced expiratory volume in 1 s (FEV_1_) and body mass index (BMI) [4–8].

The effect of VATS lobectomy in comparison to thoracotomy in reducing hospital LOS [9, 10] and postoperative pain [11] is well established. It is also becoming increasingly evident that a VATS approach may reduce incidence of PPC [10, 12–14], however, developing a PPC is still likely in patients undergoing VATS lobectomy, and PPC frequency needs confirmation, as well as identifying the effect of developing a PPC on short-term outcomes. Furthermore, few studies have specifically addressed risk factors associated with complications following VATS lobectomy, which are mainly retrospective in design, have differing definition for PPC, and only investigate the less frequent major complications [15–17].

The aims of this study were to investigate the effect of PPC on short-term outcomes after VATS lobectomy and to identify any risk factors associated with their development.

## Methods

This prospective observational study was conducted between January 2012 and January 2016 at a large single centre regional thoracic surgical unit serving six million people. Consecutive patients undergoing (VATS) lobectomy for cancer were included. Decision regarding patient operability and resectability were informed by the British Thoracic Society guidelines for lung cancer resection [18]. All patients were admitted to hospital on the day of surgery, and operations performed with single lung ventilation under general anaesthesia; patients were subsequently scheduled for extubation in the operating room.

VATS was defined as per Swanson et al. [19]; involving the use of a utility incision, without rib-spreading, two further port incisions and use of a thoracoscope to visualise the anatomical hilar dissection. Decisions regarding surgical approach by VATS rather than thoracotomy were initially conservative and based on guidance from national and international centres with extensive experience. The initial guidance included tumour size (< 7 cm), avoiding N1 involvement where known preoperatively on PET scan, no neoadjuvant chemo/radio therapy, no visibility of the tumour at bronchoscopy and no crossing of fissures by tumours. Our VATS experience has since evolved, and our cases have grown to include preoperatively identified N1 involvement, previous neoadjuvant chemo/radio therapy, visibility of the tumour at bronchoscopy requiring hand sewn bronchial stump closure and tumours crossing fissures necessitating bi-lobectomy. Exclusion criteria included re-do procedures (such as completion lobectomy) and where surgery had been indicated for pulmonary infection.

Postoperatively patients were managed in a high-dependency unit (HDU) (level 2) dedicated to thoracic surgery, and/or the thoracic surgery ward, unless the presence of complications required admission to the ITU (level 3) such as the need for invasive ventilation. The choice of analgesic technique was made by the anaesthetist after discussion with the patient. Postoperative pain control was achieved either by continuous thoracic epidural analgesia, paravertebral infusion, intrathecal morphine and/or intercostal blocks or systemic opioids (intravenous patient-controlled administration or parenteral administration).

Postoperative care also included nursing staff sitting patients out of bed on postoperative day 1 (POD1); at this point patients also started early mobilisation as able, with assistance as necessary for surgical attachments and safety. All patients were assessed by specialist thoracic surgery physiotherapists on POD1 in order to determine presence of issues amenable to physiotherapy intervention, such as atelectasis, sputum retention, or reduced mobility/ exercise tolerance. Physiotherapy treatment was then commenced as necessary in the relevant patients to clear secretions, improve lung volume or for specific mobility issues; to both increase reduced physical activity level beyond that achieved with standard care, and to regain independence. If physiotherapy was not deemed necessary patients continued with standard postoperative care. Where pulmonary complication developed physiotherapy input was escalated as appropriate. All patients received physiotherapy until resolution of pulmonary issues, and/or usual mobility independence and exercise tolerance were restored.

Data collected included demographics and preoperative record of BMI, % predicted FEV_1_, American Society of Anesthetist (ASA) score, smoking status, subjective preoperative activity level and COPD diagnosis defined by the referring clinician. Smoking data was collected by patients self-reporting to the specialist thoracic research team (including nurses and physicians) at the pre-operative assessment, and on hospital admission using a paper based case report form, which was subsequently uploaded onto the electronic database. Current smokers were defined as those who continued smoking up to the date of surgery.

Postoperative data collection included pathology reports of either primary NSCLC (staging using TNM 7th edition) or secondary metastatic disease. Hospital LOS was defined as the LOS in hospital after the date of surgery. HDU LOS and ITU admission and in-hospital mortality were also recorded. PPC was identified using a standardised scoring system named the Melbourne Group Scale (MGS), which has been previously validated by our group to define the presence of PPCs, such as pneumonia or clinically significant atelectasis [4, 20]. PPC is defined in those patients presenting with four or more of the following eight dichotomous factors: chest X-ray (CXR) findings of atelectasis or consolidation; raised white cell count (WCC) (> 11.2 × 10^9^/L); temperature > 38 °C; signs of infection on sputum microbiology; purulent sputum differing from preoperative status; oxygen saturations (SpO_2_) < 90% on room air; physician diagnosis of pneumonia; and prolonged HDU stay or readmission to HDU or ITU for respiratory complications. The MGS variables were assessed from POD1 daily by specialist physiotherapists during assessment and treatment sessions.

This study was conducted with the approval of the National Research Ethics Service (NRES) Committee West Midlands. This study was registered with the Birmingham Heartlands Hospital audit department (audit code 1672).

### Statistical analysis

Normally distributed continuous variables are expressed as mean (±SD), skewed continuous variables as median (interquartile range), and categorical variables as actual number (percentages). Normality of distributions was assessed using the Kolmogorov-Smirnov test. Differences in baseline characteristics and postoperative outcomes were analysed using Chi-square tests for categorical variables, Fisher’s exact test for categorical variables where numbers per cell were 5 or less, Independent samples t-test for continuous variables and Mann-Whitney U tests for continuous variables with skewed distributions; *p*-values < 0.05 were considered significant.

In order to determine if any factors were associated with the development of PPC tests of difference as above were performed to determine which preoperative baseline characteristics were significantly associated with PPC on univariate analysis. Any significant variables were entered into a forward stepwise logistic regression analysis to determine those independently associated with development of PPC, and to estimate the odds ratio (OR) and their 95% confidence interval (CI). Analysis was performed using IBM Statistics SPSS Version 22.

## Results

### Study population

Over the 4-year period, 291 patients underwent lobectomy using a VATS approach. Six cases were excluded; 4 were procedures undertaken for treatment of chronic lung infections and 2 were revisions or completion lobectomy. Of the 285 patients included in the study, the baseline demographics are shown in Table 1.

**Table 1:** Baseline characteristics of PPC and non-PPC patients

		Total (*n* = 285)	PPC (*n* = 21)	Non-PPC (*n* = 264)	*p* value
Gender (male)		137 (48.1%)	13 (61.9%)	124 (47%)	0.28
Age (years) median, IQR		69.0 (13)	70.0 (10)	69.0 (13)	0.84
FEV_1_% predicted mean (±SD)		87.0 (±19)	88.8 (±21.6)	87.5 (±19.8)	0.78
ASA score ≥ 3		144 (50.5%)	14 (66.7%)	130 (49.2%)	0.19
Preoperative activity level ≤ 400 m		84 (29.5%)	7 (8.4%)	77 (91.6%)	0.88
BMI ≥30		63 (22.1%)	4 (19%)	59 (22.3%)	0.49
COPD		84 (29.5%)	11 (52.4%)	73 (27.7%)	0.03
Current smoker		60 (21.1%)	9 (42.9%)	51 (19.3%)	0.02
NSCLC Staging	IA	118 (45.7%)	9 (52.9%)	109 (45.2%)	0.54
	IB	83 (32.2%)	4 (23.5%)	79 (32.8%)	
	IIA	40 (15.5%)	2 (11.8%)	38 (15.8%)	
	IIB	5 (1.9%)	0 (0%)	5 (2.1%)	
	IIIA	12 (4.7%)	2 (11.8%)	10 (4.1%)	
Secondary metastatic disease		27 (9.5%)	4 (14.8%)	23 (85.2%)	0.12
Analgesia	Paravertebral	233 (81.8%)	18 (85.7%)	215 (81.4%)	0.19
	Epidural	27 (9.5%)	0 (0%)	27 (10.2%)	
	PCA / other^a^	25 (8.8%)	3 (14.3%)	22 (8.3%)	

*PPC*, postoperative pulmonary complication; *ASA*, American Society of Anaethesiologists; *BMI*, body mass index; *COPD*, chronic obstructive pulmonary disease; *PCA*, patient controlled analgesia; ^a^morphine infusion (*n* = 3)

### Postoperative pulmonary complications

**Fig. 1:**
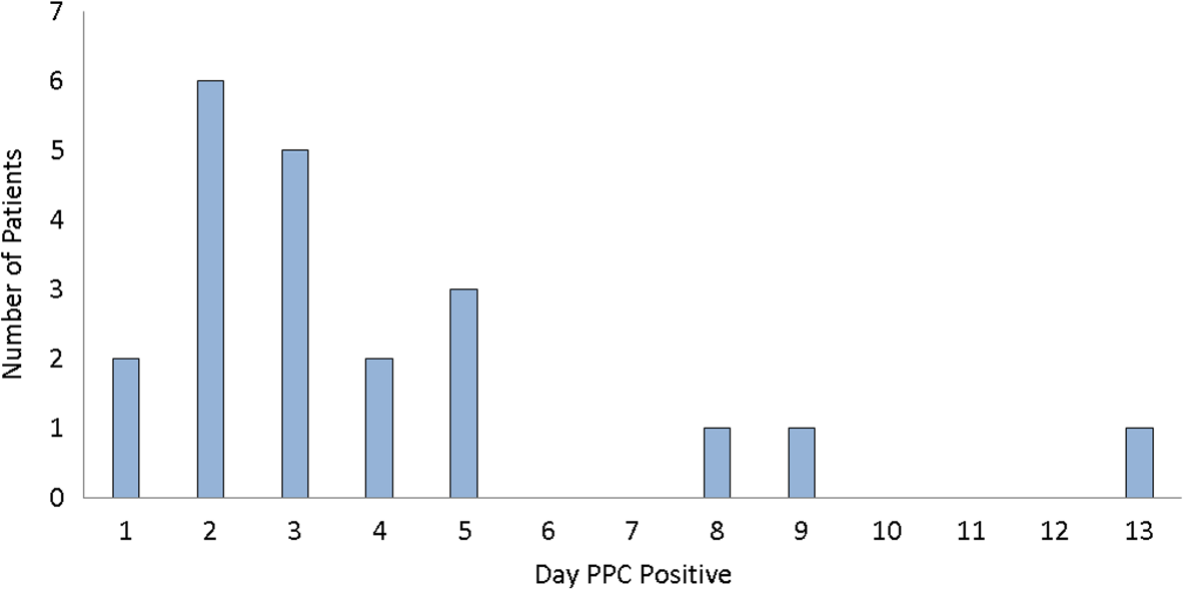
Day PPC detected following surgery

**Fig. 2:**
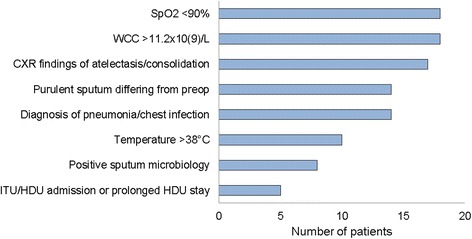
Frequency of PPC positive variables in patients who developed PPC

Twenty-one (7.4%) patients developed a PPC, most frequently (median) on POD3 (Fig. 1). Three patients developed a ‘late’ PPC, over a week after surgery (PODs 8, 9 and 13). The cases on POD8 and 9 could not be attributed as secondary to any other complication, whilst the case on POD13 was most likely related to a cerebro-vascular accident (CVA) immediately following surgery, and ensuing mechanical ventilation. The most common variables scoring positive in cases of PPC were CXR findings, elevated WCC and SpO_2_ < 90% on room air (Fig. 2).

### Outcomes

In those with PPC there was a significantly higher LOS, ITU admission and hospital mortality (*p* < 0.001) (Table 2). Of the 6 patients admitted to ITU, all developed a PPC apart from 1 patient who was admitted there following failure to wake after anaesthesia. Of the 5 patients who developed a PPC, 3 were admitted to ITU specifically because of the PPC, the other 2 developed PPC subsequent to the ITU admission. The only deaths in the study were in 3 of the 6 patients admitted to the ITU; 2 patients died secondary to a PPC, and 1 patient died secondary to a CVA.

**Table 2:** Hospital morbidity and mortality in PPC compared to non-PPC group

Outcomes	PPC (*n* = 21)	Non-PPC (*n* = 264)	*p* value
Hospital LOS (days) median (IQR)	4 (3)	3 (2)	< 0.001
ITU Admission (%)	5 (23.8%) ^a^3 (14%)	1 (0.5%)	< 0.001 0.005
Hospital mortality (%)	3 (14.3%) ^a^2 (9.5%)	0 (0%)	< 0.001 0.003

*PPC*, postoperative pulmonary complication; *ITU*, intensive treatment unit; *LOS*, length of stay; ^a^number of first mortality/ITU admission figure that were PPC related

### Early mobilisation

**Fig. 3:**
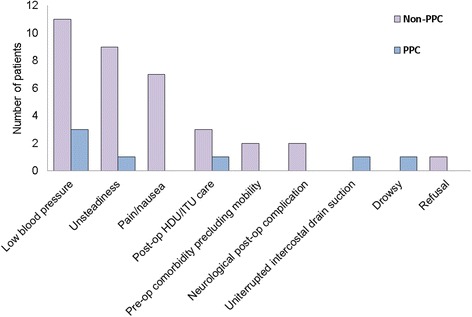
Reasons for not mobilising in POD1

Postoperative early mobilisation is shown in Table 3. Most patients (*n* = 275, 96.5%) were able to sit out of bed on POD1. In total 42 patients (14.7%) were unable to mobilise on POD 1; 10 (3.5%) were unable to sit out of bed at all and 32 (11.3%) were unable to mobilise further to their transfer out of bed. There was a significant association between reduced early mobilisation on POD1 and patients who went on to develop PPC (*n* = 7). Reasons patients were unable to mobilise on POD1 are included in Fig. 3.

**Table 3:** Early mobilisation of all patients on POD1

Early Mobility on POD1	Total (*n* = 285)	PPC (*n* = 21)	Non-PPC (*n* = 264)	*p* value
Unable to mobilise	42 (14.7%)	7 (33.3%)	35 (13.3%)	0.05
Distance Walked < 10 m	6 (2.1%)	0 (0%)	6 (2.3%)	
Distance Walked > 10 m	237 (83.2%)	14 (66.7%)	223 (84.5%)	
Unable to Sit Out^a^	10 (3.5%)	2 (9.5%)	8 (3%)	0.16

*POD1*, postoperative day 1. ^a^These patients are included in the group who were unable to mobilise

### Physiotherapy

Two hundred nine (73.6%) patients received physiotherapy treatment, with a median of 4 physiotherapy sessions in total. All 209 patients’ data showed that their physiotherapy treatment included mobilisation or exercise regardless of whether they primarily had a mobility issue or a pulmonary problem. 23 (8.1%) patients additionally required therapy to aid sputum clearance and deep breathing exercises were taught to 65 (22.8%) specifically for reduced lung expansion. Significantly more physiotherapy was required by patients who developed a PPC; they had significantly more therapist contacts and time spent on treatment (Table 4). These patients had more specific pulmonary therapies and out of hours therapy.

**Table 4:** Physiotherapy in PPC compared to non-PPC group

Physiotherapy	PPC (*n* = 21)	Non-PPC (*n* = 264)	*p* value
Need for Physiotherapy	21 (100%)	128 (48.9%)	0.002
On-call Physiotherapy (out of hours)	7 (33.3%)	4 (1.5%)	< 0.001
Number of contacts median (IQR)	10 (12)	3 (3)	< 0.001
Therapy time in minutes median (IQR)	220 (283)	70 (59)	< 0.001
Specific pulmonary interventions	16 (76.2%)	58 (22%)	< 0.001

*POD1*, postoperative day 1

### Risk factors

Univariate analysis revealed significantly higher frequency of COPD and current smokers in those with PPC (*p* < 0.05) (Table 1). Forward stepwise logistic regression was performed to determine factors independently associated with development of a PPC following VATS lobectomy. Only current smoking and COPD diagnosis were significantly more frequent in patients who had developed PPC following univariate analysis (*p* < 0.05) and were therefore included in the model. A significant contribution to the model was only made by current smoking; the OR compared to non-smokers was 3.1 (95% CI 1.3–7.8; *p* < 0.015).

## Discussion

Our study has shown a PPC frequency of 7% in cancer patients undergoing VATS lobectomy. Though this is relatively less common when compared to patients undergoing a thoracotomy approach [5, 12], we have demonstrated that PPC following VATS lobectomy is still associated with significantly worse short-term outcomes including increased ITU admission, increased hospital LOS and a higher hospital mortality. Furthermore, patients undergoing VATS lobectomy who develop PPC require more physiotherapy including sputum clearance and lung expansion therapy. Current smoking was the only significant independent factor associated with developing a PPC after VATS lobectomy.

Other studies have reported the incidence of PPC after VATS to vary between 10 to 40% [16, 17, 21], which is likely due to a lack of a standardised definition used. We have used the MGS to detect PPC such as pneumonia and clinically significant atelectasis [22], these complications have been described after VATS with an incidence of 3 to 7.5% [13, 14, 23] and 3 to 13.6% respectively [16, 21]. The MGS was initially utilised in patients undergoing thoracotomy and does not include such rare and serious postoperative complications such as broncho-pleural fistulas and pulmonary embolism. However, our study has validated its use in VATS lobectomy, and has shown that the more frequent and probably less severe PPCs detected by the MGS in these individuals are still associated with a significantly higher short-term morbidity and mortality.

The majority of patients were found to have issues potentially amenable to physiotherapy, which were mainly mobility issues; only around a quarter received physiotherapy to ameliorate specific pulmonary problems, such as atelectasis or increased/retained secretions. Additionally, patients who developed a PPC as recognised by the MGS required significantly more physiotherapy input in the postoperative period (up to three times that of other patients requiring therapy). The amount of patients requiring physiotherapy is less following VATS than thoracotomy [12], but with the frequency of mobility issues, pulmonary problems and PPC observed in this study we would recommend ‘routine’ physiotherapy assessment following VATS lobectomy, so that issues amenable to physiotherapy can be identified early.

We sought to find independent risk factors that were significantly associated with PPC following VATS. Previous studies have investigated the risk factors for PPC development after thoracotomy [4–8], but few have addressed this in VATS [15–17]. Yang et al. [17] retrospectively reported major complications that occurred in 7.3% of VATS cases for primary lung cancer, of which pulmonary complications represented 90.7% of these. The risk factors identified for major complications included age > 70 years, prolonged operation time and comorbidities including cerebrovascular disease, COPD, chronic renal insufficiency or diabetes mellitus. Interestingly, smoking was only defined as pack years (≥20) and although significant on univariate analysis (*p* = 0.002) was not significant on multivariate analysis. Wang et al. [16] studied patients (*n* = 525) who underwent VATS for lung cancer. Only the major complications occurring in 6.9% of patients were studied which including respiratory failure, haemothorax, myocardial infarction, heart failure, bronchial fistula, cerebral infarction, and pulmonary embolism. The significant independent risk factors for these major complications were age > 70 years, FEV_1_ < 70% predicted and cardiovascular disease. However, this study is limited by its retrospective nature and the extremely high proportion of never smokers (58.5%) and patients without COPD (93.5%). Our finding that FEV_1_% predicted was not predictive of PPC in VATS are support by Berry et al. [15], who found that in VATS lobectomy cases (*n* = 173) FEV_1_ was not significant independent risk factors for the 12% of patients who developed a PPC. In our study carbon monoxide lung diffusion capacity (DLCO) was performed only in patients with reduced exercise tolerance or lung volumes so data are limited, however DLCO was also found not to be a risk factor for PPC in VATS lobectomy patients [15].

To investigate the risk factors for developing PPC in patients undergoing VATS lobectomy the specific variables used in this study (age, ASA score, BMI, COPD, smoking status) were chosen as possible confounders as they had previously been shown to be independent associated with PPC following thoracotomy and lung resection in our patient group [4]. Other comorbidities such as ischaemic heart disease, heart failure, hypertension and diabetes were not previously identified as an increased risk for PPC, and therefore were not investigated in this study [5].

Our study found that smoking was the only risk factor for PPC after VATS lobectomy, which is supported by previous studies finding smoking to be the major risk factor for PPC after thoracic surgery for lung resection [4, 5]. We found that 1 in 5 patients continued to smoke up until the date of VATS lobectomy for lung cancer, and despite having being minimally invasive surgery, these patients were still 3 times more likely to develop a PPC than non-smokers. The observed effects of smoking on the increased incidence of PPC could be explained by the suppressive effect of cigarette smoking on the innate immune system. An earlier study suggested an increased risk of PPC in patients who stop smoking within 4 weeks of thoracotomy and lung resection [24], though this study was limited by its retrospective design and has since been superseded. More recent evidence has shown that the risk of PPC after thoracotomy reduces with smoking cessation, but no optimal time can be defined [25, 26]. Currently in the UK there is no integrated preoperative smoking cessation service in thoracic surgery, as only community based cessation services exist. However, these community services are designed to promote long-term quitting, which many smokers due to undergo lung cancer surgery may not be willing to commit to and most report difficulty in attending given their immediate clinical appointments; patient preference therefore is for an integrated approach [27]. We are planning to undertake a feasibility study to investigate if personalised intense smoking cessation intervention integrated into the thoracic surgical pathway improves smoking cessation rates when compared to usual care of standard community based NHS smoking cessation.

### Study strength and limitations

This is the first prospective observational study to investigate the risk factors associated with the development of PPC in patients undergoing VATS lobectomy. One limitation of this study is that smoking status was self-reported by patients prior to surgery, and although patients tend to under-report smoking status, biochemical confirmation would need to confirm this in future studies. Another limitation of the study is that the protocol of postoperative analgesia was not the same in all patients. For example, none of the patients who developed a PPC had an epidural catheter. Despite this, we have previously demonstrated in a large group of thoracic surgery patients that choice of analgesia was not a risk factor for the development of PPC after lung resection [5]. Finally, we recognise a limitation to the findings of the study with the small number of patients who developed PPC within the regression model; in a larger cohort it is possible that other risk factors may have been identified. However, the lower frequency of PPC following VATS lobectomy precludes the observation of a large group in a timely manner.

## Conclusions

Despite the minimally invasive nature of surgery patients undergoing VATS lobectomy for cancer remain at risk of developing a PPC, which is associated with significantly worse short-term morbidity and mortality. Physiotherapy was applied in most patients to ameliorate mobility or pulmonary issues, however, those developing a recognised PPC required significantly more treatment. Current smoking is an independent risk factor for PPC following VATS lobectomy, thus vigorous addressing of preoperative smoking cessation is urgently needed.

## Abbreviations

ASA: American Society of Anaethesiologists
BMI: body mass index
CI: confidence interval
COPD: chronic obstructive pulmonary disease
CVA: cerebro-vascular accident (CVA)
CXR: chest x-ray
FEV_1_: percentage predicted forced expiratory volume in one second
HDU: high-dependency unit
ITU: intensive therapy unit
LOS: length of stay
MGS: Melbourne Group Scale
NSCLC: non-small cell lung cancer
OR: odds ratio
POD1: postoperative day 1
PPC: postoperative pulmonary complication
VATS: video-assisted thoracoscopic surgery
WCC: white cell count
